# Adaptation trajectories during adhesion and spreading affect future cell states

**DOI:** 10.1038/s41598-017-12467-4

**Published:** 2017-09-26

**Authors:** Stéphanie M. C. Bruekers, Min Bao, José M. A. Hendriks, Klaas W. Mulder, Wilhelm T. S. Huck

**Affiliations:** 1 Radboud University Nijmegen, Institute for Molecules and Materials, Heyendaalseweg 135, 6525 AJ Nijmegen, The Netherlands; 2 Radboud University, Radboud Institute for Molecular Life Sciences, Geert Grooteplein 28, 6525 GA Nijmegen, The Netherlands

## Abstract

Cells are complex systems in which dynamic gene expression and protein-interaction networks adapt to changes in the environment. Seeding and subsequent spreading of cells on substrates represents an example of adaptation to a major perturbation. The formation of adhesive interactions and self-organisation of the cytoskeleton during initial spreading might prime future cell behaviour. To elucidate the role of these events on later cellular behaviour, we mapped the trajectories by which cells respond to seeding on substrates with different physical properties. Our experiments on cell spreading dynamics on collagen- or fibrin-coated polyacrylamide gels and collagen or fibrin hydrogels show that on each substrate, cells follow distinct trajectories of morphological changes, culminating in fundamentally different cell states as quantified by RNA-expression levels, YAP/TAZ localisation, proliferation and differentiation propensities. The continuous adaptation of the cell to environmental cues leaves traces due to differential cellular organisation and gene expression profiles, blurring correlations between a particular physical property and cellular phenotype.

## Introduction

Cells are complex systems, where the interplay of many (simple) components leads to the emergence of highly sophisticated behaviour^1^. The cellular state at a particular time can be characterised by the combined abundance and organisation of all its components. A key challenge is to understand how cells reach a particular state upon a response to changes in their environment^2^. As a first step, one can study the isolated components, including gene expression levels and protein localisation at steady. However, cell development is a dynamic process, following trajectories across a metaphorical ‘landscape’ of gene expression profiles that involve multiple so-called attractors^3,4^. Changes in the environment will affect this ‘landscape’, as the cell adapts by altering cellular organisation and changing gene expression profiles, thus potentially altering cell state and cell fate. An important example of such an adaptation process is the spreading of cells on a substrate, the dynamics of which have been studied in detail^5–10^. It is clear that adaptation to the substrate, and the forces experienced during spreading, lead to different dynamic changes in cell shape^11,12^. The balance of forces, the development of focal adhesions, and the build-up of tension in the cytoskeleton on substrates with different mechanical characteristics, have all been captured in impressive studies^11–15^. With time, cells reach a steady state, and numerous studies have demonstrated correlations between a wide range of mechanical characteristics and steady state properties such as cell adhesion, spreading area, proliferation and differentiation^16–23^. The question we address here, is whether the adaptation of cellular shape and organisation during the spreading of cells on substrates with different mechanical properties, impacts on future cellular phenotypes and cell fate. We therefore developed a time-resolved, systems level study, which would allow us to follow both invariant and divergent characteristics of cells while they spread on different substrates, and provide a direct window on the cellular processes that integrate the multitude of mechanical cues over time.

Here, we follow how hMSCs adapt, upon seeding, to different substrates (PAAm hydrogels coated with collagen and fibrin vs. collagen and fibrin hydrogels) over 24 hours. On each substrate, cells follow distinct trajectories of morphological changes, culminating in fundamentally different cell states, as reflected in significant differences in gene expression profiles and protein localisation characteristics. These results challenge the view that characterisation of cellular phenotypes at apparent steady states without knowledge of the prior events can provide us with a complete picture of how cells sense the mechanical properties of their environment.

## Results

Human mesenchymal stem cells (hMSCs) were cultured on polyacrylamide (PAAm) gels of medium (≈3 kPa) and high (≈23 kPa) stiffness, coated with either collagen filaments or fibrin monomers and compared to hMSCs cultured on collagen type I (<1 kPa) or fibrin (<1 kPa) gels, respectively (see materials and methods for a detailed description of the formation of substrates and Figures S1 and S2 for the characterisation). These PAAm vs. protein substrates differ in mechanical properties (stiffness, strain stiffening, porosity) but are as similar as possible in the biochemical cues they present.

**Figure 1:**
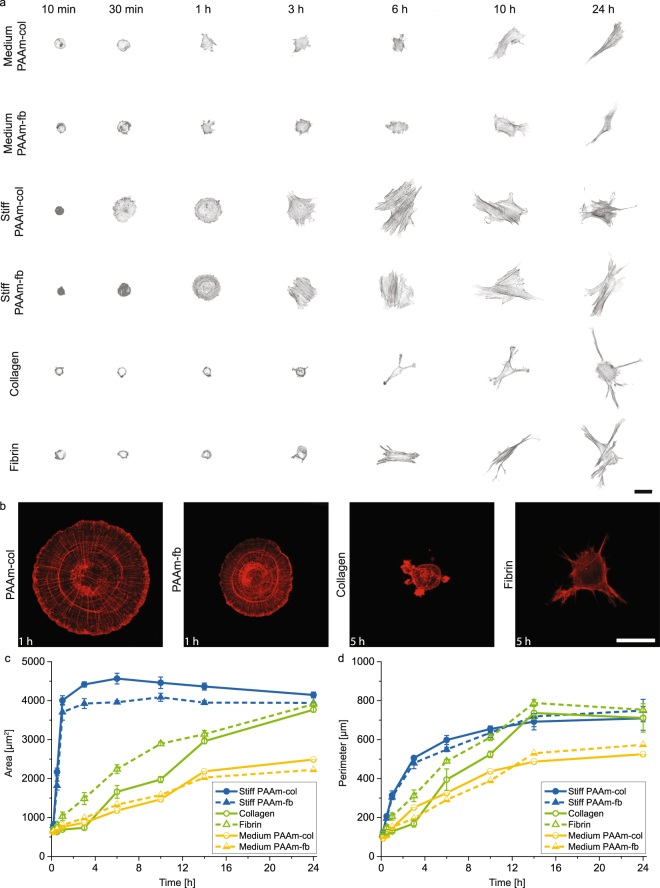
Cell spreading dynamics is very different for PAAm and protein gels (**a**) hMSC spreading at time points that illustrate the route of spreading on the six gel types. Representative cells were selected for each time point. Scale bar 50 µm. (**b**) Actin structure during the different trajectories of spreading showing the radial and transverse fibres after 1 h on the stiff PAAm gels and the onset of spreading with the formation of protrusions after 5 h on the protein gels. Scale bar 50 µm. (**c**) Quantification of cell area and (**d**) perimeter over time, showing the different spreading trajectories on PAAm and protein gels. Mean ± SEM from three replicates (mean from two for medium PAAm) with over 50 cells analysed per sample per replicate, ANOVA oneway analysis followed by Tukey *post hoc* correction per timepoint is shown in Tables S1 and S2.

We followed hMSC adhesion and spreading from seeding up to 24 hours (morphology-wise considered a ‘steady state’ in the field) using live cell imaging techniques. Low cell densities were used in order to observe single cells and eliminate cell-cell communication. Figure 1a and Movies S1–S4 show representative cells in different stages of spreading and remarkable differences were observed between the spreading on the coated PAAm hydrogels of different stiffness compared to the protein hydrogels. The initial spreading of cells on stiff PAAm gels was highly isotropic and cells adopted a striking disc-like morphology. The actin cytoskeleton had a radial arrangement as well as multiple transverse fibres which together appeared as circular actin rings (Fig. 1b)^24^. This radial and transverse fibre organisation was transient and eventually small protrusions appeared. The cells then adopted more irregular shapes (Fig. 1a) showing parallel actin stress fibres, as commonly observed^15,25,26^. In contrast, cells on protein gels remained small and we observed the formation of protrusions after 30 min up to several hours after seeding (Fig. 1a,b). An evident increase in cell area occurred only at a later stage, in which the cells adopted a well spread morphology with actin fibres present mainly in the protrusions of the cell body. Finally, cells on PAAm gels of medium stiffness (≈3 kPa) exhibited markedly smaller spreading areas over the course of 24 h.

**Figure 2:**
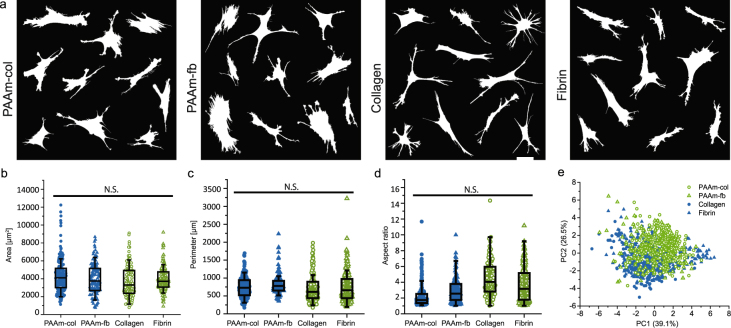
hMSC spreading on stiff PAAm and protein gels shows only minor differences between the gels after 24 h (**a**) Cell outlines of ten representative cells per gel type. Scale bar 50 μm. (**b**) Cell area, (**b**) Cell area, (**c**) Cell perimeter, (**d**) Aspect ratio distribution from triplicate experiments with a minimum of 60 cells per sample and (**e**) Principal component analysis for cells on different substrates. Mean ± SEM, ANOVA oneway analysis shows significance levels of p > 0.05.

The evolution of cell morphology on all six substrates is shown in Fig. 1c,d. The increases in both cell area and cell perimeter show distinct trajectories for cells on each of the different substrates. However, as can already be seen from the convergence of some of the trajectories in Fig. 1c and d, the morphology after 24 h can be much more similar, especially if one compares the cells on fibrin gels with those on fibrin-coated stiff PAAm (Fig. 2a–d). We investigated the morphology of cells on the different substrates at this time point in more detail. For this, we extracted 11 quantitative morphological features (eg. area, perimeter, roundness) from hundreds of cells seeded on protein hydrogels and coated stiff PAAm substrates (Figure S3). Principal component analysis was used to uncover features, or combinations of features, that can separate the cells from the different substrates. We found that the top 7 principal components explain >95% of the variation in the data. However, none of these clearly separate the different substrates from each other. This highlights the convergence of cell morphologies over time to reach a steady state 24 hours after seeding the cells (Fig. 2e).

**Figure 3:**
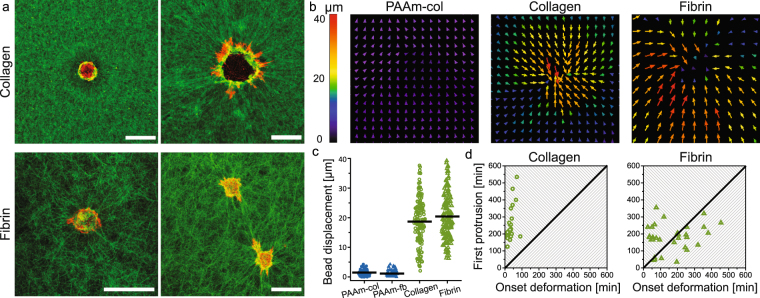
Gel deformation and cell spreading over time: (**a**) Round (left) and spread (right) hMSCs stained for actin (red) on labelled collagen and fibrin gels (green). Both round and spread cells can deform the matrix. All scale bars represent 50 µm. (**b**) Gel deformation was quantitatively measured by bead displacement. The cells on PAAm gels do barely deform the matrix, whereas the cells on collagen and fibrin can deform the gel over long distances. (**c**) Average displacement of beads within 150 µm of cells on PAAm-col, PAAm-fb, collagen or fibrin. (**d**) Comparison between onset of deformation and spreading. On collagen, all cells first deform the matrix before they started to spread (top left part). On fibrin, approximately half of the cells first deformed the matrix, the others only deformed the matrix after spreading (bottom right part).

The combination of distinct spreading trajectories but significant overlap in morphology at 24 h prompted us to explore the spreading trajectories on stiff (≈23 kPa) PAAm and protein gels in more detail. We observed that cell spreading on protein gels is associated with cellular remodelling of the substrates (shown by live cell imaging experiments, Movies S3 and S4, and by imaging fluorescently labelled hydrogels (Figs 3a and S4). Fibre recruitment has been described as a mechanism by which cells probe and respond to mechanics in fibrillar matrices^11,26^, and our findings are in agreement with previous literature reports^27–30^. We performed bead displacement studies to quantitatively compare substrate deformation on PAAm and protein substrates (Movies S5–S7). Figure 3b and c shows representative images of the displacement fields on stiff PAAm, collagen and fibrin gels. On PAAm gels of different stiffness, very small bead displacements were measured (average 1.5 ± 0.7 µm for stiff PAAm), showing that cells barely deform the PAAm gels. As fibre recruitment has been associated with matrix stiffening of protein hydrogels^28,31^, we used AFM indentation measurements to characterise cell-induced stiffening of the gel before the onset of spreading. Figure S5 clearly shows regions of increased stiffness in the protein matrix surrounding cells. As stiff PAAm gels are providing a rigid substrate, the cells can form pronounced focal complexes. On the protein gels, focal complexes were observed only occasionally, and these were less pronounced and smaller than on the PAAm substrates which is consistent with previous literature (Figure S6)^32^. The average onset of bead displacement (quantified from 32 cells) was approx. 30 min on collagen and 4 hours on fibrin. After 8.5 h the motion of the fluorescent beads ceased, indicating mechanical equilibrium between the cell traction forces and the elastic resistance of the protein gels, possibily in combination with remodelling of the gels^26,33,34^. Within this time frame, most cells remained within the window of recording. Figure 3d shows how the onset of deformation and onset of spreading are related for individual cells. All cells on collagen gels deformed the matrix before protrusions were observed. On fibrin this process seemed to be more dispersed, with a similar fraction of cells first deforming the matrix, or first spreading. We cannot exclude that there are small, short-lived protrusions that we were unable to observe causing the deformations. Nevertheless, a difference in cell spreading remains between collagen *versus* fibrin as the onset of deformation is much sooner on collagen and cell area increases earlier on fibrin than on collagen.

It is evident that cell spreading follows distinct trajectories associated with different materials properties of the substrate. We wished to investigate the intracellular imprints of these trajectories by studying the changes in gene expression over time using genome-wide RNA sequencing to obtain more molecular insight into the dynamics of cellular processes during the observed dynamic spreading behaviour on the different types of substrates. For this, we harvested cells for RNA isolation after 1 h, 5 h, 10 h and 24 h and compared expression levels to the original cell suspension at the moment of seeding as a common reference sample in technical duplicates (t_0_A and t_0_B). We chose to compare RNA expression levels on collagen and fibrin gels vs. stiff PAAm gels coated with collagen and fibrin, as these substrates showed different trajectories but closest morphologies after 24 h. See SI for more experimental details. Interestingly, the number of differentially expressed genes increases much faster on protein gels compared to PAAm gels, even though changes in morphology occur much faster on PAAm, see Figure S7.

**Figure 4:**
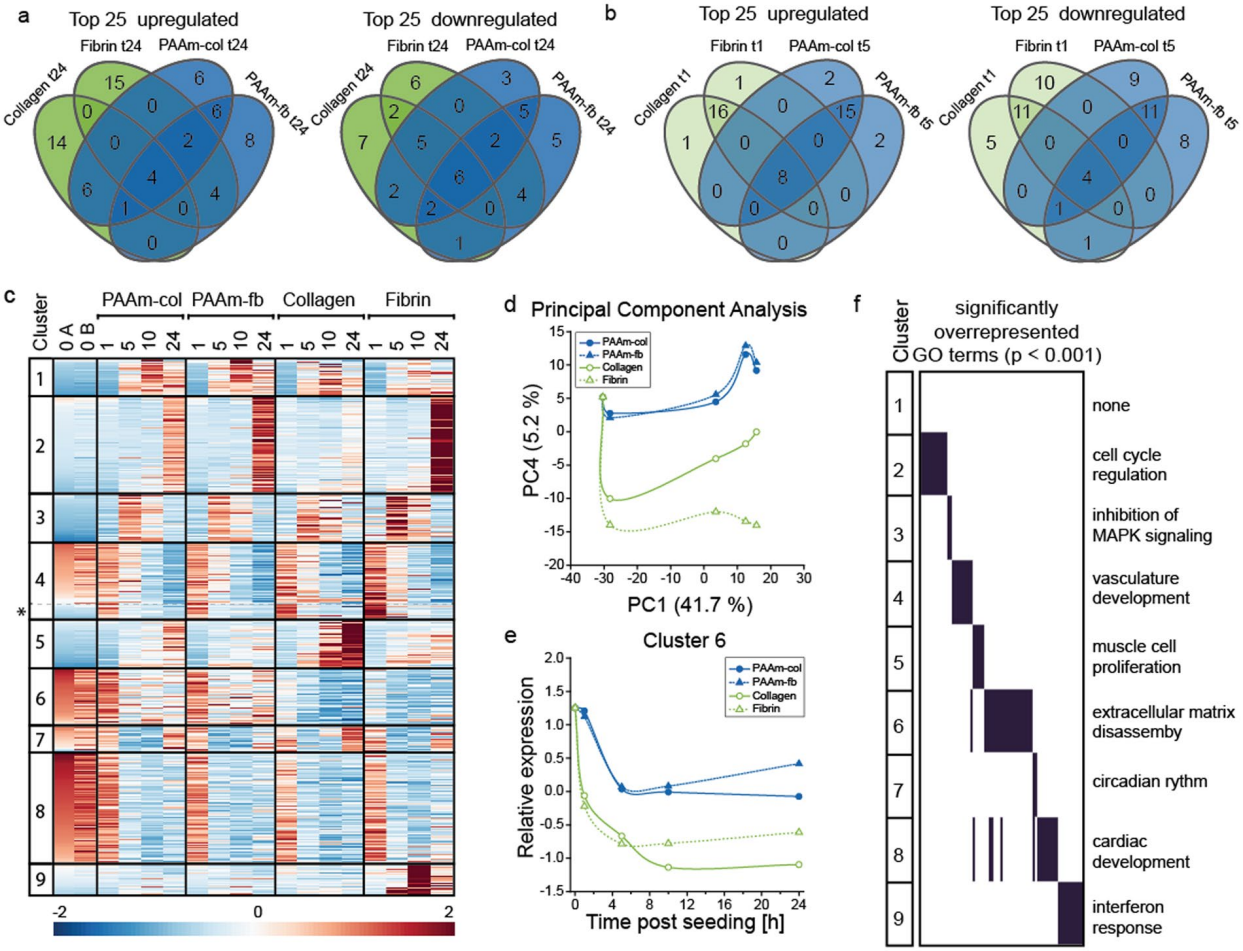
Differences in dynamic gene expression programs associate with observed cell spreading behaviour and highlights underlying mechanisms (**a**) Venn diagrams summarising the magnitude of the overlap in top 25 upregulated (left) and downregulated (right) genes per substrate after 24 h showing the divergence in RNA-expression profiles. (**b**) Venn diagrams summarising the magnitude of the overlap in top 25 upregulated (left) and downregulated (right) genes per substrate after 1 h at the protein gels and 5 h at the PAAm gels indicating that the difference in number of differentially expressed genes after 1 h is not simply delayed on PAAm gels compared to the protein gels. (**c**) Heat map representation of the Z-scores of gene expression levels clustered by K-means clustering (**d**) Principal Component 4 can distinguish the PAAm substrates from the protein gels (**e**) Average relative expression levels from genes in cluster 6 show differences in gene expression on PAAm and protein gels (**f**) Selection of significantly overrepresented GO terms (p < 0.001) per cluster.

We focussed on 948 RNA transcripts that were reliably detected at t_0_ (fragments per kilobase of exon per million reads mapped (FPKM) ≥ 2) and were differentially expressed in at least one time-point compared to t_0_ (p ≤ 10^−10^ and fold-change ≥5). For a general overview on similarities and differences between samples at identical time points, the top 25 upregulated and downregulated genes were compared. The overlap in the top 25 upregulated genes between the various substrates decreased over time (Figure 4a,b), indicating divergence in cell state in time on the four types of substrates. After 24 h, clear differences between fibrin and collagen gels were observed for top 25 upregulated genes, whereas cells on PAAm-col and PAAm-fb gels showed somewhat more overlap (Fig. 4a). No clear trend was observed in the top 25 downregulated genes after 24 h. To investigate whether the difference between protein and PAAm gels in the number of differentially expressed genes (Figure S7) is a delay or an indication for a different response, we also compared the top 25 genes on the protein gels after 1 h to the PAAm gels after 5 h. From the top 25 upregulated and downregulated genes, only 8 and 4 genes, respectively, were present in this selection on all four gel types (Fig. 4b, central numbers). This clearly indicates that changes in gene expression are not simply delayed on the PAAm substrates.

For further and more detailed analysis, K-means clustering (9 clusters) was applied (top-bottom) for all 18 samples (left-right, Fig. 4c). Several clusters show dynamic gene regulation over time that is consistent among all four substrates. For instance, a subsection of Cluster 4 (denoted 4*) includes genes, including FOSB and JUNB, that are upregulated one hour after seeding and subsequently downregulated. Similarly, Cluster 1 and Cluster 3 (including FOS and JUN targets) show similar transient upregulation with a slightly delayed onset (Figure S8). Genes in these three clusters show essentially identical patterns on all four substrates. The similarity between the different substrates indicates that similar networks of immediate early genes^35^ are active in cells on all substrates in the early stages upon seeding.

K-means clustering analysis also identified several clusters that showed differences in gene expression between PAAm and protein gels (Cluster 6), as well as substrate specific effects (clusters 2, 5 and 9). We used principal component analysis (PCA) to identify common underlying structures in the RNA-seq dataset that could help identify the observed differences between the samples (Fig. 4d). Principal component 1 (PC1, explaining 41.7% of the variability of the dataset) essentially orders all the samples according to time (Fig. 4d). In contrast, PC4 (5.2%) distinguishes the PAAm substrates from the protein gels (Fig. 4b) and with the genes in Cluster 6 being the key contributor to this difference (Fig. 4e).

To identify the biological and molecular processes represented in the different clusters, we performed gene ontology overrepresentation (GO) analysis. A selection of the biological processes that were found significantly overrepresented (p < 0.001) are presented in Fig. 4f, a full overview can be found in Table S3. Analysis indicated that Cluster 6, which distinguishes PAAm from protein gels, seems to be enriched in genes involved in extracellular matrix turnover. Examples are ACAN, FBN1, FBN2, HSPG2, COL11A1, COL12A1, COL14A1, COL25A1 and COL4A1. These genes are coding for the proteins aggrecan, fibrillin, heparin sulfate proteoglycan 2 and several collagens, which are all components of the ECM. This indicates that the cells on PAAm gels are producing more ECM proteins and hence are modifying their environment. These are important differences, although a detailed analysis of how these sets of genes alter the ‘internal’ trajectories is beyond the scope of this study.

**Figure 5:**
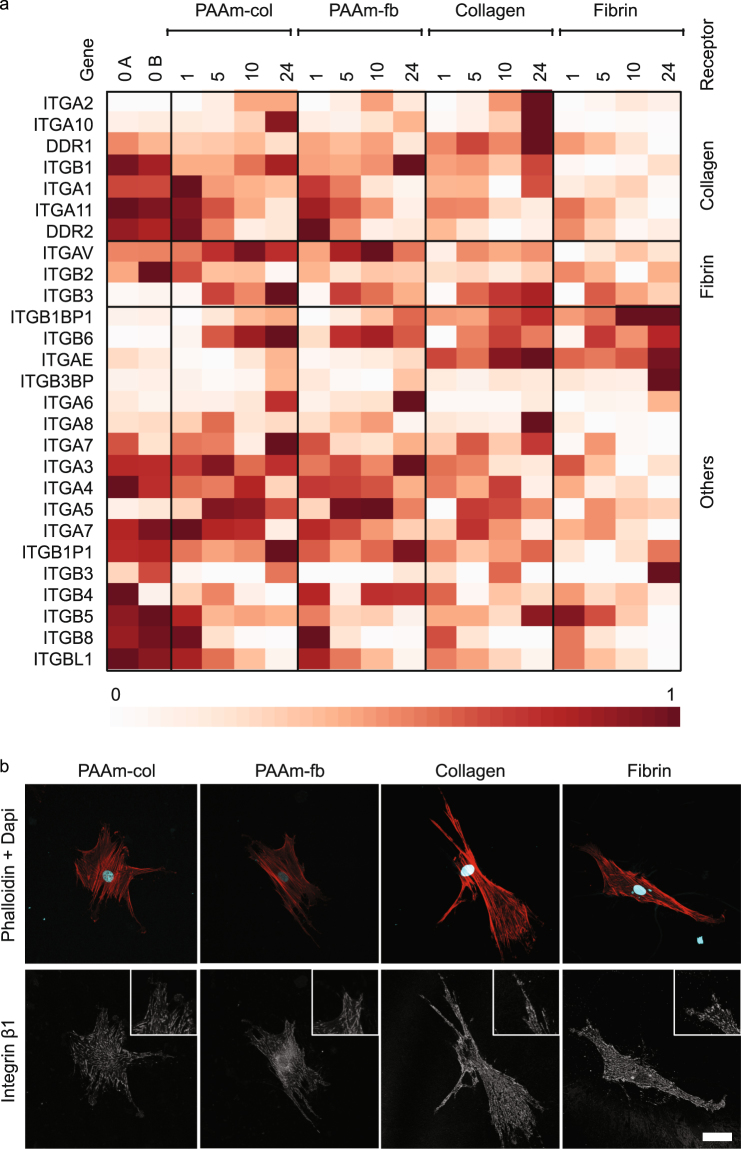
Differences and similarities in cell adhesion to the substrates (**a**) Heat map representation of gene expression levels (normalised scale) of cell adhesion receptors that were present on all samples and time points. (**b**) Integrin β1 adhesions are present on all gels after 24 h. Insets contain enlarged details of Integrin β1 adhesions. scale bar 50 µm.

Our RNA-seq data also provided information on integrin expression levels on the different substrates. Integrins provide the physical linkage between the ECM and the cytoskeleton and play an important role in mechanotransduction^36–38^. Figure 5a provides an overview of all integrin subunits as well as discoidin domain receptors that were expressed on all samples and all time points (FPKM > 0). This heat map clearly shows that cells express different adhesion molecules on the various substrates. Integrin β1, a principal receptor for collagen, is highly expressed on all substrates (FPKM > 10^3^), albeit slightly lower on fibrin gels. Immunostainings showed that activated integrin β1 is present on all substrates and aligned with intracellular stress fibres (Fig. 5b). Other collagen-binding integrins show more distinct expression levels on the different substrates. The expression of Integrin α2, typically associated with collagen binding, increases in time on collagen substrates, but decreases after 10 h on the other three gel types. Integrin α10 expression increased on all substrates except fibrin gels. Interestingly, we also observed that discoidin domain receptor family member 1 (DDR1), a protein involved in the communication of cells with their microenvironment, showed increasing expression levels on collagen gels, while slightly decreasing on the other substrates. Fibrin-binding and other integrins show various other differences between the substrates. Interestingly, there is no clear difference between PAAm-col and collagen gels on one hand and PAAm-fb and fibrin gels on the other, the total pattern of integrins that is expressed seems dependent on more factors than only the cell type used and the adhesion molecules present. Furthermore, this subsection of genes again shows that cells on each substrate follow distinct trajectories.

**Figure 6:**
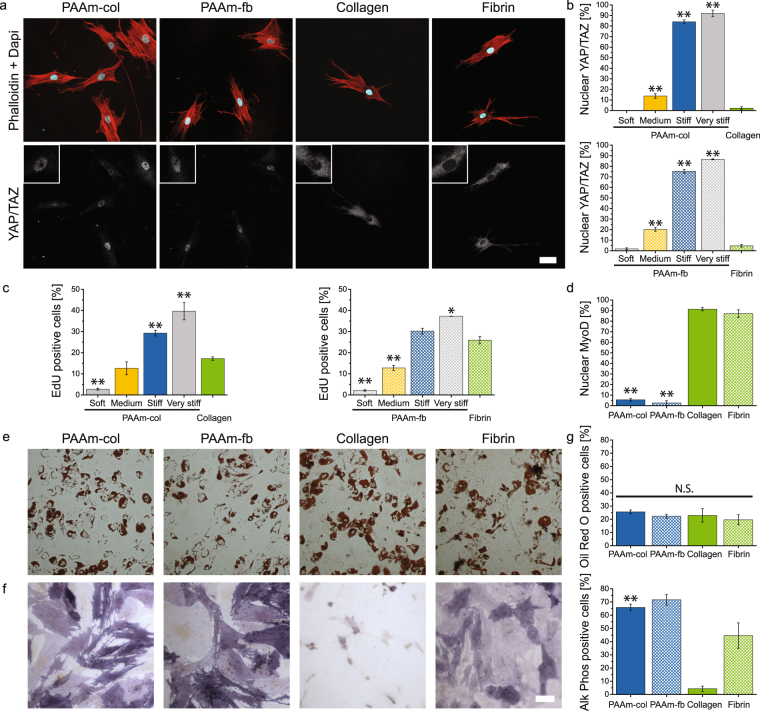
Different gene-expression dynamics indicate long-term outcomes of cellular state (**a**) YAP/TAZ localisation in hMSCs on the four gel types after 24 h. Insets contain enlarged details of nuclear YAP/TAZ localization. Scale bar 50 µm (**b**) Quantification of YAP/TAZ nuclear localisation on different PAAm stiffness (soft ≈1 kPa, medium ≈3 kPa, stiff ≈23 kPa and very stiff ≈1.1 × 10^2^ kPa) with our standard stiff PAAm gel in blue in comparison with the protein gels in green. Collagen and collagen coated gels in homogeneous colours, fibrin and fibrin coated gels striped columns. (**c**) Cell proliferation determined by EdU incorporation over 24 h. (**d**) Quantification of nuclear localisation of MYOD (**e**) Oil Red O and (**f**) Alkaline Phosphatase staining on the four gel types. Scale bar 100 µm. (**g**) Quantification of differentiation after 7 d (Alk Phos) and 10 d (Oil Red O). Mean ± SEM, ANOVA oneway analysis followed by Tukey *post hoc* test shows significance levels of *p < 0.05, **p < 0.01 and NS: p > 0.05 compared to corresponding collagen or fibrin gel.

As a final set of experiments, we wished to confirm that the differences between substrates we observed in morphology or in gene expression levels, are also present at a protein level. In recent studies, cell spreading on stiff substrates has been associated with nuclear localisation of two downstream components of the Hippo pathway: Yes-associated protein (YAP) and transcriptional coactivator with PDZ-binding motif (TAZ). It is thought that cells growing on stiff substrates can develop stress fibres and cytoskeletal tension, which are known to prevent phosphorylation of YAP/TAZ, resulting in nuclear localisation^39^. As TEAD1, a transcription factor activated by the mechanosensitive YAP1^39–42^, was among the genes in Cluster 6, we tested whether the type of substrate could have an effect on YAP/TAZ localisation in hMSCs after 24 h of culture. Fluorescence staining (Fig. 6a) shows that YAP and TAZ remain located in the cytosol of the cells on collagen and fibrin. Quantification of triplicate experiments show nuclear localisation in 2.3 ± 2.1% of hMSCs on collagen and 4.7 ± 2.1% on fibrin gels (Fig. 6b). Control experiments on PAAm gels showed that on stiff gels there was indeed nuclear localisation (75–85% on stiff PAAm and 10–20% on medium PAAm) and YAP/TAZ remained in the cytosol on soft PAAm (approximately 2% nuclear localisation, Fig. 6b). Similar to cells on very soft hyaluronic acid gels^43^, but in contrast to the majority of existing literature, cell spreading and F-actin organisation alone are not sufficient to cause YAP and TAZ to localise into the nucleus. It should be noted that YAP/TAZ localisation is not simply delayed in cells cultured on protein gels: we found that after culturing the cells for 48 h on the protein gels, still no nuclear localisation occurred (Figure S9).

Interestingly, Cluster 2 is enriched in genes involved in cell cycle progression and proliferation, whereas Cluster 5 is enriched in muscle cell proliferative genes. Thus, our RNA-sequencing analysis suggests that cells seeded on collagen only gels might initiate towards a muscle cell fate and do not follow the same cell proliferation program as cells on the other substrates. Subsequent quantification of hMSC proliferation as determined by EdU incorporation over 24 h shows significantly lower proliferation on collagen than on stiff PAAm-col whereas cell proliferation on fibrin is not significantly different from cells on stiff PAAm-fb (Figs 6c and S10). Additionally, cells cultured on collagen and fibrin were found to have nuclear localisation of MyoD after 24 h of culture, indicating their potential differentiation towards muscle (Figs 6d and S11)^44^.

Finally, we wished to explore how the different trajectories in the first 24 hours (both in terms of morphology as in RNA expression levels and protein localisation) impacted on future events, such as the differentiation of hMSCs into osteoblasts and adipocytes^15,21,45–47^. hMSCs were seeded on the four types of gels. After culturing in mixed adipogenic/osteogenic medium for 10 days, Oil red O staining showed no difference between the substrates in the relative presence of adipocytes (Fig. 6e). Differentiation into osteoblasts was examined after culturing the hMSCs for 7 days in mixed medium. On PAAm and fibrin gels, approximately 60–70% and 40%, respectively, of cells stained positive for Alkaline Phosphatase. Cells on collagen gels, however, did not differentiate towards osteoblasts (Fig. 6f,g). These experiment again illustrate that initial differences in interactions between cells and their substrate lead to long-lasting differences in cell behaviour.

## Conclusions

We have shown that cellular adaptation to the mechanical environment upon seeding follows distinct trajectories. Differences in phenotypes arise early, as exemplified by the isotropic spreading on stiff PAAm gels, whereas cells on protein gels show matrix deformation, and spreading along deformed regions. However, after strikingly different initial morphologies, cellular phenotypes appear to converge over time, and after 24 h cells can look remarkably similar. In contrast, the morphological trajectories are accompanied by intracellular ones that do not converge: RNA-seq data show on the one hand that cells on all substrates follow a common expression pattern of early response genes, but differences appear in for example integrin expression patterns, especially when comparing PAAm and protein gels, but also between collagen and fibrin substrates. At 24 h, the ‘molecular starting points’ for further development, i.e. the occupancy of the complex networks that govern cell behaviour and responses, are very different among the different substrates. Gene expression profiles do show that the early differences have resulted in different molecular trajectories, leading cells to integrate the various mechanical cues in different ways. The different trajectories also lead to significant differences in the localisation of YAP/TAZ, proliferation rates, and cellular differentiation on different substrates. It is clear that the different adaptation routes cells take on different substrates leave an imprint, and affect future events such as lineage selection.

Our studies make clear that the continuously evolving patterns of morphological changes and gene expression profiles that result from probing the environment, blur the correlations between a particular property of the substrate and cellular phenotype. The impact of different environmental factors such as matrix elasticity or topography might thus depend on how they shape the adaptation trajectory that cells take. Our findings highlight the importance of studying the response of cells over time, and early spreading trajectories could provide a strong indication of future cellular behaviour.

## Materials and Methods

### Preparation of polyacrylamide hydrogels

This method was based on a previously described protocol^6^. Glass coverslips (13 mm, thickness no 1, borosilicate glass) were oxidised using oxygen plasma and then incubated in a 0.3 wt/vol% solution of 3-(trimethoxysilyl)propyl methacrylate (Sigma Aldrich) in dry toluene overnight. The slides were washed thoroughly with ethanol and water.

Solutions of acrylamide (AA) at final concentrations of 5, 8 and 20 wt/vol% and bis-acrylamide (BA) at 0.01, 0.02, 0.15 and 0.375 wt/vol% were prepared. Polymerisation was initiated by the addition of 5 µL of 10 wt/vol% ammonium persulfate (Sigma Aldrich) and 1.5 µL TEMED (Sigma Aldrich) to the AA/BA solutions in PBS. 4 µL of the gel precursor solution was immediately pipetted onto de methacrylated glass coverslips and a 20 mm glass coverslip, washed but untreated, was carefully placed on top of the polymerizing solution. After 1.5–2 h, the samples were soaked in PBS buffer overnight to remove the remaining monomer and crosslinker. The top coverslips were peeled off to obtain the PAAm gels adhering to the coverslides.

To facilitate cell adhesion, the hydrogel was covered with a covalently bound protein coating using N-sulfosuccinimidyl-6-(4′-azido-2′-nitrophenylamino)hexanoate (sulfo-SANPAH, Life Technologies) as a crosslinker. 2 × 15 µL of a 1 mg mL^−1^ solution in milliQ H_2_O was pipetted onto the gel surfaces, which were then placed under a 365 nM UV lamp (ABM, USA) and irradiated for 5 min. The gels were washed twice with PBS and the procedure was repeated once. After the second round of washing with PBS, the substrates were coated with 50 µg mL^−1^ of rat type I collagen in PBS for 2 h at room temperature or with 0.1 mg mL^−1^ of bovine fibrinogen in PBS for 1.5 h followed by 0.5 h incubation with thrombin 1 U mL^−1^ in PBS. Samples were again washed two times with PBS prior to cell seeding.

### Preparation of fibrin hydrogels

Fibrinogen (FBNG, from bovine plasma, Sigma Aldrich) was dissolved in PBS (4 mg mL^−1^) and then put through a 0.22 µm filter to sterilise the solution. Gelation was achieved by mixing the FBNG solution with CO_2_-independent medium (DMEM-Hepes, 10% FBS) in equal volumes, resulting in 2 mg mL^−1^ fibrin gels after 1 h incubation at 37 °C. For gel deformation studies, fibrin gels were stained directly after gelation with FITC prior to cell seeding.

### Preparation of collagen hydrogels

Rat tail collagen type I (BD biosciences) gels were prepared similarly to the manufacturer’s protocol. High concentration collagen was diluted to 4 mg mL^−1^ with pre-mixed 1/10 of the volume with 10x PBS, 0.023x the collagen volume of 1 M NaOH was added to neutralise the pH. An equal amount of DMEM-Hepes was added to dilute the collagen to a 2 mg mL^−1^ solution. Gelation occurred during 30 min incubation at 37 °C. For gel deformation studies, collagen gels were stained directly after gelation with primary anti-collagen antibody (Abcam, ab34710) for 1 hour at 37 °C and subsequently with Alexa488-conjugated secondary antibody (Thermo Fisher, A-11001), prior to cell seeding.

### Characterisation of the substrates

Substrate stiffness was measured by nanoindentation under an atomic force microscope (Bruker Nanoscope) using the “point and shoot” procedure (Nanoscope software, Bruker). A fluorescent polystyrene bead (φ = 10 μm, Invitrogen) was glued to silicon nitride cantilevers with nominal spring constants of 0.06 N/m (NP-S type D, Bruker). The system was calibrated in cell-free medium at 37 °C prior to each experiment by measuring the deflection sensitivity when pressing the cantilever onto a glass coverslip, which allowed the cantilever spring constant to be determined using the thermal noise method^38^. For each gel, indentation force curves at 30 different locations on the gels were acquired. Before and during indentation experiments gels were kept in medium in 37 °C. To address local stiffness changes generated by cells on protein gels and PAAm gel, we applied spatially resolved AFM nanoindentation in live-cell culture by probing the matrix around single cells. To obtain stiffness values from force curves we used the PUNIAS software (http://punias.free.fr). Specifically, we corrected for baseline tilt, and used the linear fitting option for the Hertz model with a Poisson ratio of 0.5 on the indentation curve.

### Culturing of hMSCs and seeding onto substrates

hMSCs were obtained from Lonza and cultured up to passage 6 before seeding them onto gels at a density of circa 1250 cm^−2^ for the analysis of spreading, gel deformation and YAP/TAZ expression. Spreading and gel deformation were observed at multiple time points. YAP/TAZ expression was studied after 24 and 48 h in culture.

All adipo/osteo differentiation experiments were performed in a 1:1 mixture of adipogenic and osteogenic induction medium (DMEM + 10% FBS + pen/strep, containing 5 × 10^−7^ M dexamethasone, 5 mM β-glycerolphosphate, 0.1 mM ascorbic acid-2-phosphate, 250 µM 3-isobutyl-1-methylxanthine, 5 µg mL^−1^ insulin (from bovine pancreas) and 5 × 10^−8^ M rosiglitazone maleate). Cells were seeded at circa 2500 cm^−2^ for osteogenic differentiation and 25000 cm^−2^ for adipogenic differentiation in MIX medium on the substrates. Medium was changed two times per week. Osteogenic differentiation was analysed after 7 days by staining for alkaline phosphatase, and adipogenic differentiation after 10 days by staining with Oil red O. ALP/Oil red O positive cells were counted manually from at least five different bright field images. The percentage of differentiation towards each lineage was quantified by setting a uniform threshold in each experiment and manually counting the number of positively stained cells in relation to the total number of nuclei (DAPI staining).

### Immunofluorescence staining

hMSCs on hydrogels were fixed with 4% PFA for 10 min and permeabilised with 0.2% Triton-X100 for 10 min. Following blocking with 10% BSA solution in PBS for 1 h, substrates were incubated with primary antibodies (vinculin (Abcam, ab18058), YAP/TAZ (Cell signalling, D24E4), β1 integrin (BD Biosciences, 550531), MyoD (BD Biosciences, 554130) and Alexa633-conjugated phalloidin (Sigma Aldrich) and subsequently with Alexa488 or -546-conjugated secondary antibodies (Life Technologies) and DAPI. For proliferation studies, EdU labelling was performed following the manufacturer’s protocol (Click-iT EdU Alexa Fluor-488 HCS Aaasy, Thermo fisher scientific). Quantification of nuclear localisation was performed manually. Cells were considered to have nuclear localisation when the level of fluorescence in the nucleus was higher than the level in the cytosol.

### Live cell imaging

Phase contrast images were acquired at 2 min intervals on an IncuCyte live cell analysis system (Essen BioScience). For bead displacement studies, PAAm pre-gels as well as collagen and fibrin solutions were mixed with fluorescent beads, which served as fiduciary markers for tracking the deformation of the matrix under adherent cells. The bead displacement field around each cell was mapped from a time series of fluorescent images that were collected using an inverted time lapse microscope (Nikon Diaphot 300 with Hamamatsu C8484-05G CCD Camera, Okolab CO_2_ stage incubator and Okolab 2D time lapse software). The initial bead position was determined ~20 min after cell seeding, when cells were attached to the gel surface. A time series of images was collected at a frequency of one image every 10 min. Beads displacement analyses were performed with a custom script in Fiji software.

### RNA isolation and purification

RNAs from 6 subsamples were isolated by an extraction using Trizol reagent (Life Technologies) according to the manufacturer’s protocol. The RNA pellets from these subsamples were resuspended in water and pooled to a total volume of exactly 100 µL. The samples were further purified by means of a Nucleospin RNA clean-up kit (Machery-Nagel), including an on column treatment with rDNAse. Finally the samples were eluted in 60 µL of RNAse-free water. Quality control was performed using an Agilent 6000 nano kit on an Agilent BioAnalyzer 2100. Subsequent RNA sequencing was performed by ServiceXS Leiden, The Netherlands. For the reference samples at t_0_ a technical duplicate was performed (A and B) by using two separate aliquots of the cell suspension prior to seeding on the substrate.

### Statistical analysis

Significance of differences throughout this study were tested using one-way analysis of variance (ANOVA) with Tukey *post hoc* correction with *p < 0.05, **p < 0.01 and NS: p > 0.05. Analysis was performed on triplicate independent experiments using cells from the same donor throughout. ANOVA was chosen over t-tests due to its more conservative nature.

## Electronic supplementary material

MovieS1

MovieS2

MovieS3

MovieS4

MovieS5

MovieS6

MovieS7

Supplementary Data File (Table S3)

Supplementary Figures and Tables
