# Supplementary material for: Adaptation trajectories during adhesion and spreading affect future cell states
Source: Sci Rep. 2017 Sep 26;7:12308. doi: 10.1038/s41598-017-12467-4 (PMC5615062; doi:10.1038/s41598-017-12467-4)
Supplement: Supplementary file 9 — Supplementary Figures and Tables [file 41598_2017_12467_MOESM9_ESM.docx]

**Adaptation trajectories during adhesion and spreading affect future cell states**

Stéphanie M. C. Bruekers^1†^, Min Bao^1†^, José M. A. Hendriks^1^, Klaas W. Mulder^2^ and Wilhelm T. S. Huck^1^*

**Supplementary information**


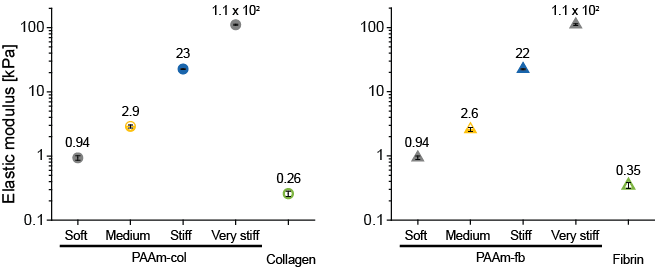


Figure S1: **Gel characterisation by AFM** showing the elastic modulus of all gels used.


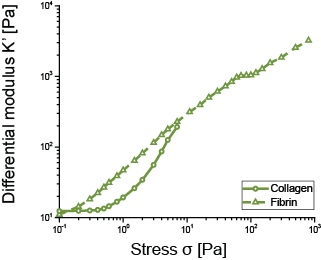


Figure S2: **Gel characterisation by rheology** showing the strain-stiffening response of the protein gels.


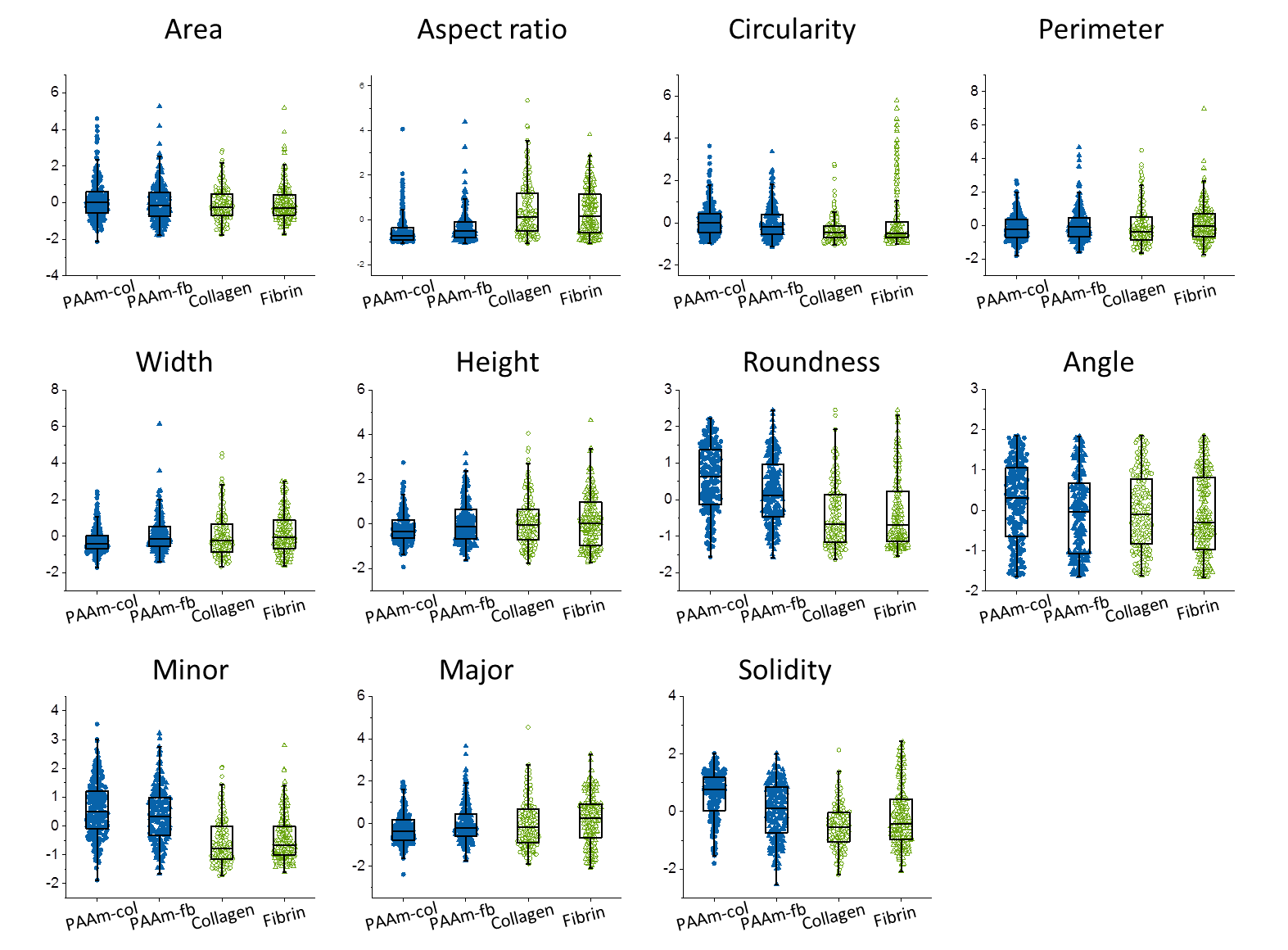


Figure S3: 11 quantitative morphological features (eg. area, aspet ratio, circularity, perimeter, roundness) from hundreds of cells seeded on protein hydrogels and coated stiff PAAm substrates. Morphological features were extracted by a custom plugin in Fiji software.


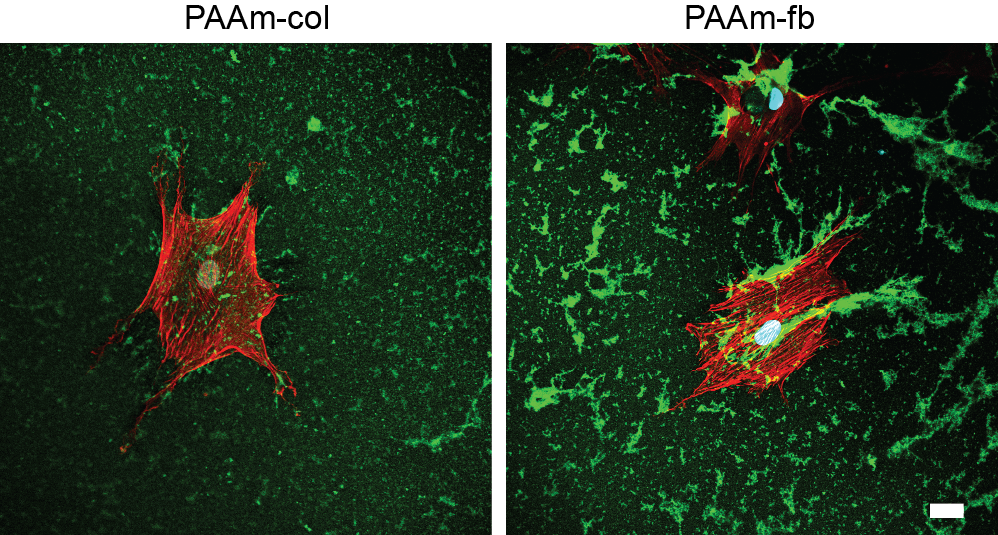


Figure S4: hMSCs stained for actin (red) and nucleus (blue) on PAAm gels stained with collagen (left) and fibrin monomer (right). Both collagen and fibrin monomer were homogenously coated on PAAm hydrogels. Scale bar 10 µm.


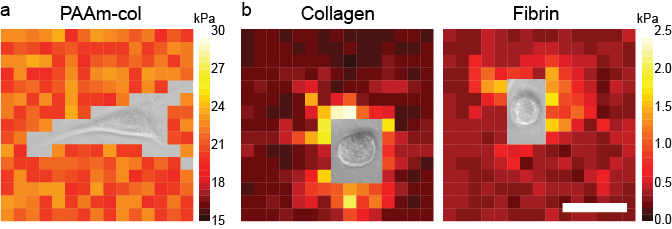


Figure S5: **Gel stiffening after cell adhesion** The elastic modulus of the gels around the cells was mapped using AFM and showing (a) no change in modulus around cells on PAAm and (b) an increased stiffness around round cells on the protein gels after 3 h of culture. Scale bar 50 µm.


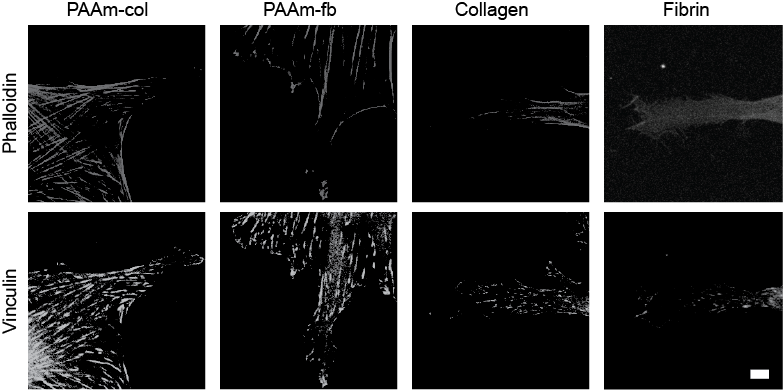


Figure S6: **Focal adhesions are larger and more pronounced on the PAAm gels** Vinculin staining after 24 h culture. Scale bar 10 µm.


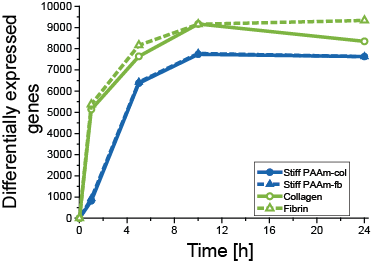


Figure S7: **Total number of differentially expressed genes per gel type over time** These numbers are very similar on PAAm-col and PAAm-fb, causing the lines to overlap. After 1 h there are many more genes differentially expressed on the protein gels compared to the PAAm gels


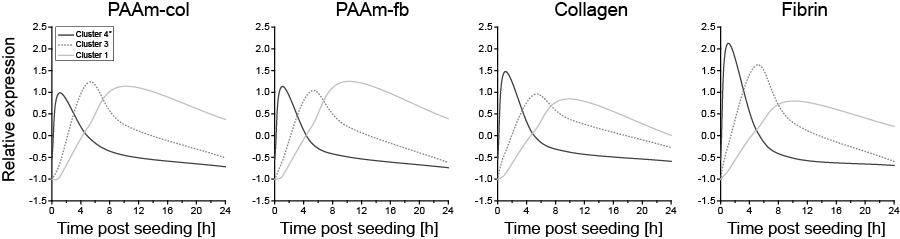


Figure S8: **Transient gene upregulation that is similar on all gels** Average Z scores show that the kinetics of expression sequences are similar on the different substrates


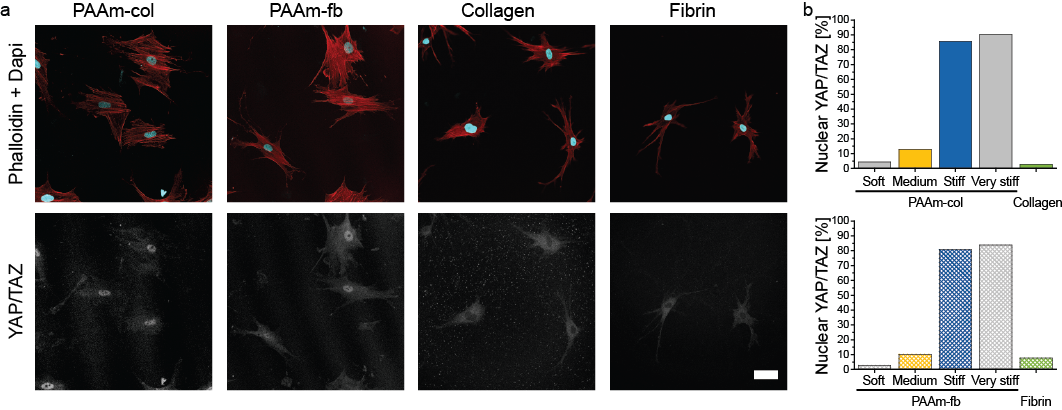


Figure S9: **No nuclear YAP/TAZ on protein gels after 48 h** (a) YAP/TAZ localisation in hMSCs on the four gel types after 48 h. Scale bar 50 µm (b) Quantification of YAP/TAZ nuclear localisation on different PAAm stiffness with our standard PAAm gel in blue in comparison with the protein gels in green. Collagen and collagen coated gels in homogeneous colours, fibrin and fibrin coated gels striped columns.


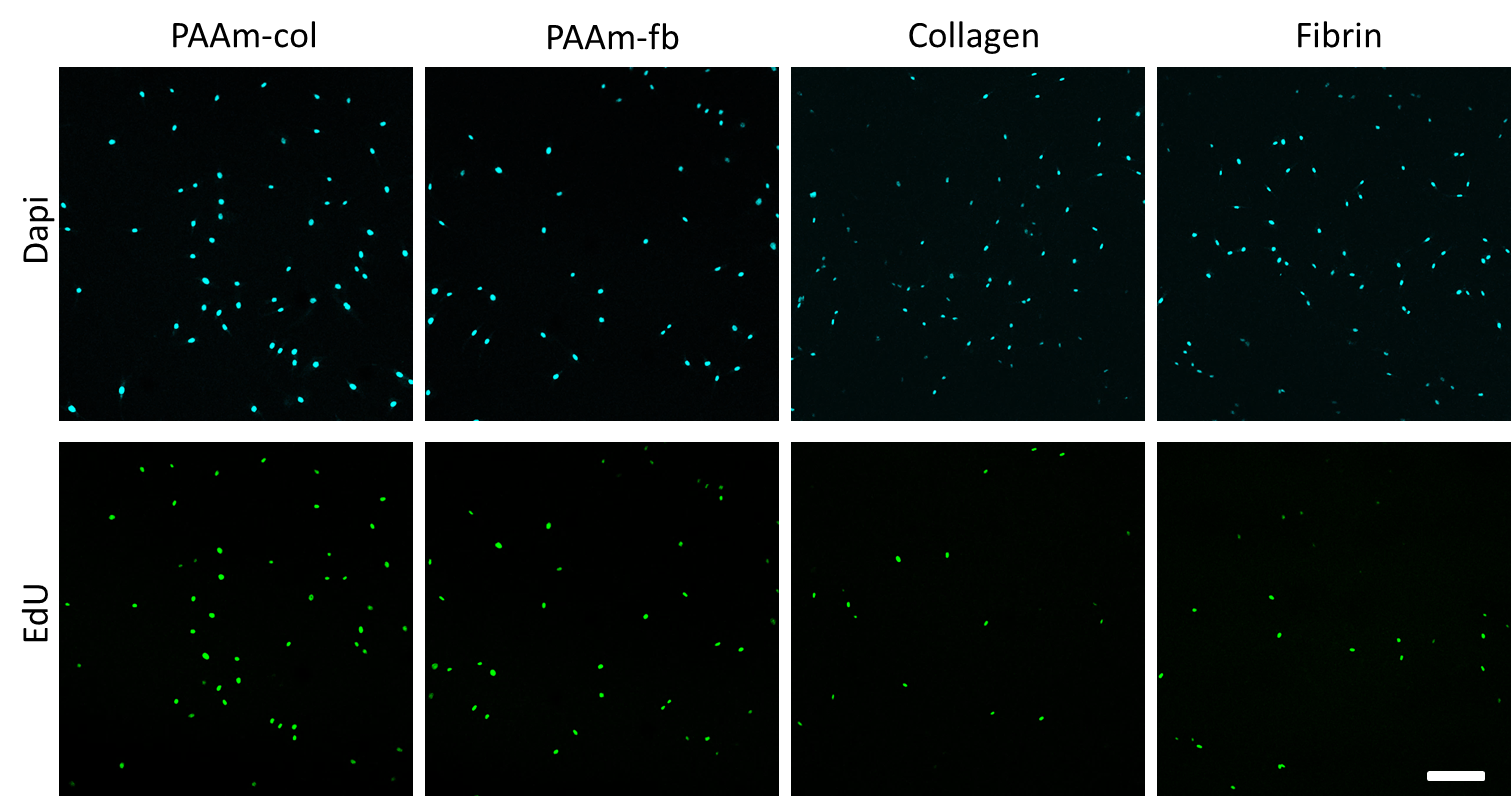


Figure S10: **Cell proliferation determined by EdU incorporation.** Representative EDU staining images for cells cultured on PAAm-col, PAAm-fb, collagen and fibrin after 24 hours. Scale bar 200 µm.


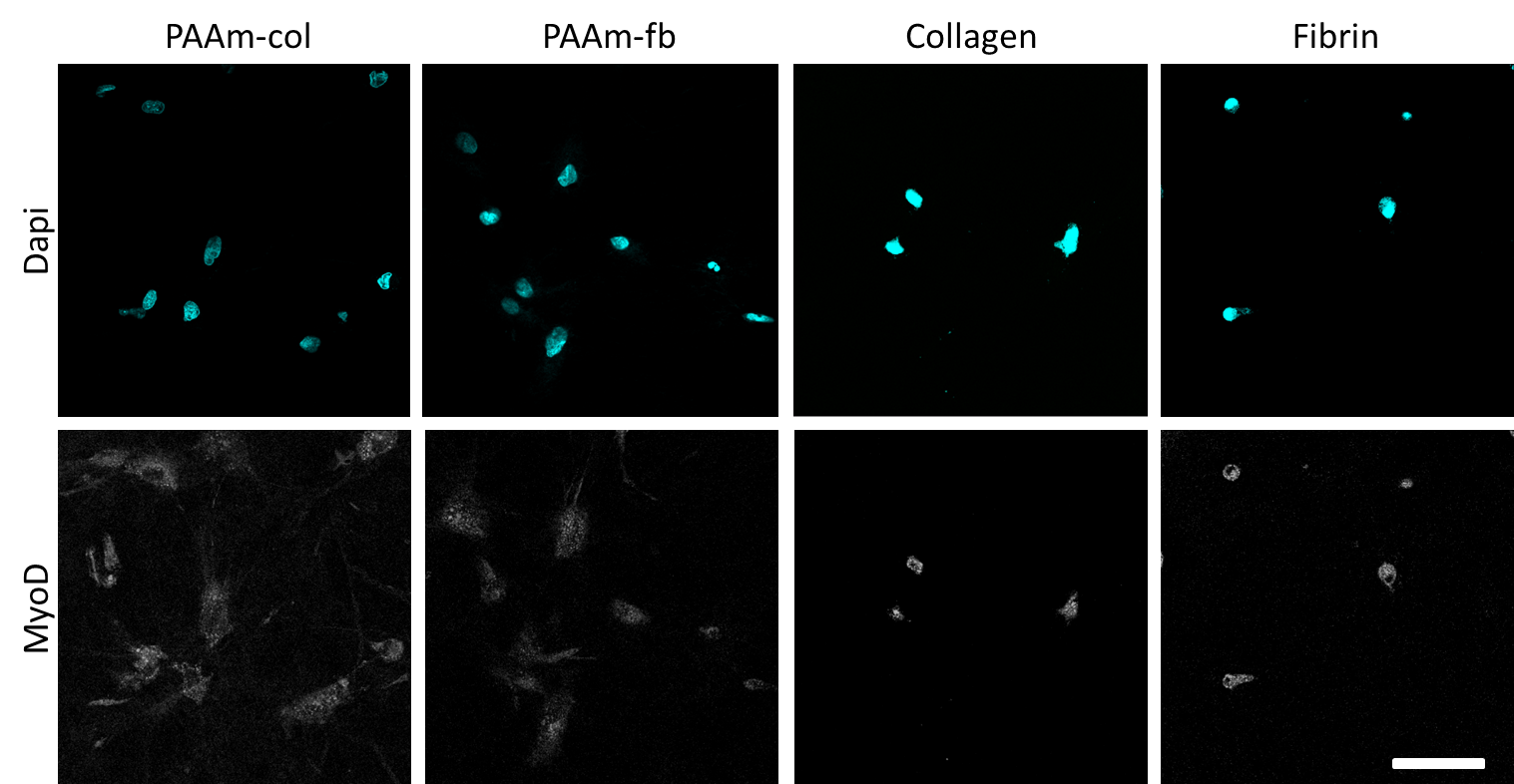


Figure S11: **Cells on protein gels have potential differentiation towards muscle.** Representative MyoD staining images for cells cultured on PAAm-col, PAAm-fb, collagen and fibrin after 24 hours. cells cultured on collagen and fibrin were found to have nuclear localisation of MyoD. Scale bar 200 µm.

Table S1: **Statistical analysis of cell area over time per time point**

| Timepoint |  | Stiff PAAm-Col vs collagen | Stiff PAAm-fb vs fibrin |
| --- | --- | --- | --- |
| 10 min |  | NS | NS |
| 30 min |  | ** | ** |
| 1 h |  | ** | ** |
| 3 h |  | ** | ** |
| 6 h |  | ** | ** |
| 10 h |  | ** | ** |
| 14 h |  | ** | * |
| 24 h |  | NS | NS |

NS: p>0.05, *: p<0.05, **: p<0.01

Table S2: **Statistical analysis of cell perimeter over time per time point**

| Timepoint |  | Stiff PAAm-Col vs collagen | Stiff PAAm-fb vs fibrin |
| --- | --- | --- | --- |
| 10 min |  | NS | NS |
| 30 min |  | ** | * |
| 1 h |  | ** | NS |
| 3 h |  | ** | NS |
| 6 h |  | NS | NS |
| 10 h |  | NS | NS |
| 14 h |  | NS | NS |
| 24 h |  | NS | NS |

NS: p>0.05, *: p<0.05, **: p<0.01

Table S3: **Overview of overrepresented GO terms**: separate Excel file

Movie S1: **Live-cell imaging of hMSC spreading on PAAm-col**: separate Avi file

Movie S2: **Live-cell imaging of hMSC spreading on PAAm-fb**: separate Avi file

Movie S3: **Live-cell imaging of hMSC spreading on collagen**: separate Avi file

Movie S4: **Live-cell imaging of hMSC spreading on fibrin**: separate Avi file

Movie S5: **Bead displacement of hMSC spreading on PAAm-col**: separate Avi file

Movie S6: **Bead displacement of hMSC spreading on collagen**: separate Avi file

Movie S7: **Bead displacement of hMSC spreading on fibrin**: separate Avi file
